# The external ear morphology and presence of tragi in Australian marsupials

**DOI:** 10.1002/ece3.6634

**Published:** 2020-08-28

**Authors:** Hayley J. Stannard, Kathryn Dennington, Julie M. Old

**Affiliations:** ^1^ School of Animal and Veterinary Sciences Charles Sturt University Wagga Wagga NSW Australia; ^2^ School of Science and Health Hawkesbury Campus Western Sydney University Penrith NSW Australia

**Keywords:** Dasyuridae, diet, insectivore, mammal, pinnae

## Abstract

Multiple studies have described the anatomy and function of the external ear (pinna) of bats, and other placental mammals, however, studies of marsupial pinna are largely absent. In bats, the tragus appears to be especially important for locating and capturing insect prey. In this study, we aimed to investigate the pinnae of Australian marsupials, with a focus on the presence/absence of tragi and how they may relate to diet. We investigated 23 Australian marsupial species with varying diets. The pinnae measurements (scapha width, scapha length) and tragi (where present) were measured. The interaural distance and body length were also recorded for each individual. Results indicated that all nectarivorous, carnivorous, and insectivorous species had tragi with the exception of the insectivorous striped possum (*Dactylopsila trivirgata*), numbat (*Myrmecobius fasciatus*), and nectarivorous sugar glider (*Petaurus breviceps*). No herbivorous or omnivorous species had tragi. Based on the findings in this study, and those conducted on placental mammals, we suggest marsupials use tragi in a similar way to placentals to locate and target insectivorous prey. The Tasmanian devil (*Sarcophilus harrisii*) displayed the largest interaural distance that likely aids in better localization and origin of noise associated with prey detection. In contrast, the smallest interaural distance was exhibited by a macropod. Previous studies have suggested the hearing of macropods is especially adapted to detect warnings of predators made by conspecifics. While the data in this study demonstrate a diversity in pinnae among marsupials, including presence and absence of tragi, it suggests that there is a correlation between pinna structure and diet choice among marsupials. A future study should investigate a larger number of individuals and species and include marsupials from Papua New Guinea, and Central and South America as a comparison.

## Graphical abstract

We investigated the pinnae of marsupials, with a focus on the presence/absence of tragi and how it may relate to diet. Of the 23 Australian marsupial species studied, only nectarivorous, carnivorous, and insectivorous species had tragi. The results suggest a correlation between pinnae structure and diet choice among marsupials.

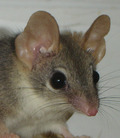

## 1 INTRODUCTION

The sensory system of each animal has evolved and adapted to meet their unique requirements. Auditory perception, or hearing, is essential to the survival of most species. It ranges from a rudimentary capacity to feel vibrations in arthropods and reptiles (Bennet‐Clark, 1971; Christensen, Christensen‐Dalsgaard, Brandt, & Madsen, 2012) to the complex determination of airborne sound waves in mammals (Borg & Engstrom, 1983). Hearing in therians evolved and was refined during the Triassic Period in the earliest mammals (Manley, 2017). Hearing in mammals is essential for interspecies communication, and in predator‐prey relationships where it aids recognition, defence and avoidance of predators, as well as prey localization (Aitkin, Nelson, & Shepherd, 1994; Apfelbach, Blanchard, Blanchard, Hayes, & McGregor, 2005; Brechin, Wilshusen, Fortwangler, & West, 2002; Ratcliffe, Fullard, Arthur, & Hoy, 2011; Wang, Li, Li, & Zhang, 2011).

There is a strong selection pressure on hearing with respect to predator and prey relationships. Failing to avoid a predator or catch prey can result in detrimental consequences for the individual (Jones, Holloway, Ketcham, & Long, 2008). It is therefore likely that both mammalian predator and prey species have been subjected to strong selection pressure during the evolution of their hearing structures to aid either predator avoidance or prey capture. Aitkin (1997) defined the ‘acoustic biotope’ as the concept that species will be adapted to the sounds that they are likely to naturally encounter in their environment, such as predator/prey, conspecifics and abiotic sounds of wind and water. Diversity of a species' acoustic biotope is a result of environments differing through space and time. The term refers specifically to natural noises that the species perceive (Johannesma & Aertsen, 1982). Well‐adapted species have a hearing range that encompasses their acoustic biotope—hence, organisms through natural selection will, over time, have developed better hearing in relation to their environment (Aitkin et al., 1994). The frequency and amplitude that each species is capable of hearing therefore can vary greatly. Humans can hear a range of 2‐20 kHz, while guinea pigs (*Cavia porcellus*) can hear up to 40 kHz and some bat species over 70 kHz (Manley, 2017). These hearing range discrepancies are correlated with differences in the size and stiffness of the middle ear (Nummela & Sanchez‐Villagra, 2006), and the external ear (pinna) supports hearing, specifically aiding sound capture and channeling of the sound to the tympanic membrane through the external auditory meatus (Purves et al., 2001).

Excluding placental (subfamilies Talpinae and Scalopinae) and marsupial moles (*Notoryctes* spp.) and monotremes, all terrestrial mammalian species have visible pinnae (Aitkin, 1997). The pinnae amplifies and transfers information (through sound reflection) to the middle and inner ear for interpretation by the brain (Hayward, Jędrzejewski, & Jêdrzejewska, 2012; Rosowski, 1996). Removal experiments, such as those conducted on the insectivorous brown long‐eared bat (*Plecotus auritus*) (Muller, Lu, & Buck, 2008), that has large prominent pinnae, have provided information about the performance of information intake by the pinnae. Muller et al. (2008) confirmed that the pinnae of brown long‐eared bats are vital in providing the species with directional and spatial information on its surroundings. Features involved with the form and function of pinnae, such as the interaural distance, relates to the frequency detection of large mammals (Heffner & Heffner, 1998), while the tragi, a structure located at the entrance to the external auditory meatus, and mostly studied in insectivorous bats relates to noise localization acuity (Koay, Kearns, Heffner, & Heffner, 1998). The manipulation of the tragus by Aytekin, Grassi, Sahota, and Moss (2004) determined that prey capture performance of the big brown bat (*Eptesicus fuscus*) lowered significantly when compared to the control group. Hence, by moving the pinnae, some nose‐leaf bats can control the amplitude of noises and position their ears to deduce soundwave emission locations (Kuc, 2010), a feature required to capture insectivorous prey successfully, that may be utilized by other species to capture insect prey.

When compared to placentals, research on marsupial hearing and pinnae anatomy is limited (Aitkin, 1995; Aitkin et al., 1994; Cone‐Wesson, Hill, & Liu, 1997; Gates & Aitkin, 1982; Old, Tulk, & Parsons, 2020; Reimer, 1995). It is, however, important to explore auditory perception in marsupials and investigate its vital role in predator evasion, prey identification, and interspecies communication. This study investigated the comparative anatomy of the pinnae in a range of Australian marsupials, specifically the presence/absence of tragi and correlated it to diet. Insectivorous bats have prominent tragi (Aytekin et al., 2004), and we aimed to investigate whether carnivorous/insectivorous marsupials likewise had prominent tragi. This correlation may lend support to the hypothesis that the tragus aids insect prey location and acquisition in marsupials.

## 2 MATERIALS AND METHODS

The species incorporated in this study included as many Australian marsupial families as possible but was limited to preserved specimens available for study at the Australian Museum, Sydney, NSW. Individual specimens were selected based on being preserved with the ear upright or out, and with the majority of tissue retained to ensure accuracy in pinna measurements. Mostly adult fully grown specimens were selected, and individuals with ears that showed notches or excessive damage were excluded. In addition, no specimens of Notoryctemorphia (marsupial moles) were included in this study as they lacked visually discernible pinnae.

Measurements recorded included scapha length and width of the pinna, and the interaural distance from the left ear to the right ear. The presence or absence of tragi was noted, and where present the horizontal width of the tragus was measured. Measurements were taken using digital Vernier callipers, with exception of specimens that were too large, and instead, a ruler was used. Body length (from the tip of nose to where the tail joins the lower spinal column), body mass (if noted before preservation methods), sex, and tag ID number and collection location were also recorded for each specimen. Specimens were not weighed post‐preservation as this would have been an inaccurate representation of their live body weight.

### 2.1 Data analysis

The species studied were categorized into diet groups based on the main dietary items of each species using Hume (2006) and Woinarski, Burbidge, and Harrison (2014). We categorized animals into the following diet groups: insectivores, carnivores, omnivores, herbivores, and nectarivores. A phylogenetic tree showing the relationship between the major clades of marsupials was adapted from May‐Collado, Kilpatrick, and Agnarsson (2015). The tree was pruned and matched to the dataset analyzed using R packages *caper*, *ape,* and *geiger* (Harmon, Weir, Brock, Glor, & Challenger, 2008; Orme et al., 2018; Paradis & Schliep, 2019). Generalized least squares fit models were used to determine associations between scapha length, scapha width, width of the tragus, and interaural with body length using log‐transformed data, similar to analyses used by Weisbecker, Speck, and Baker (2019). We computed PGLS analyses using the R packages *nlme* and *ape* (Paradis & Schliep, 2019; Pinheiro, Bates, DebRoy, & Sarkar, 2020). We plotted the data points and means for each species using scatter plots in RStudio. An ANOVA with LSD post hoc tests was used to determine differences between measurements and diet type, in SPSS (IBM Corporation).

## 3 RESULTS

### 3.1 Ear morphology

**FIGURE 1:**
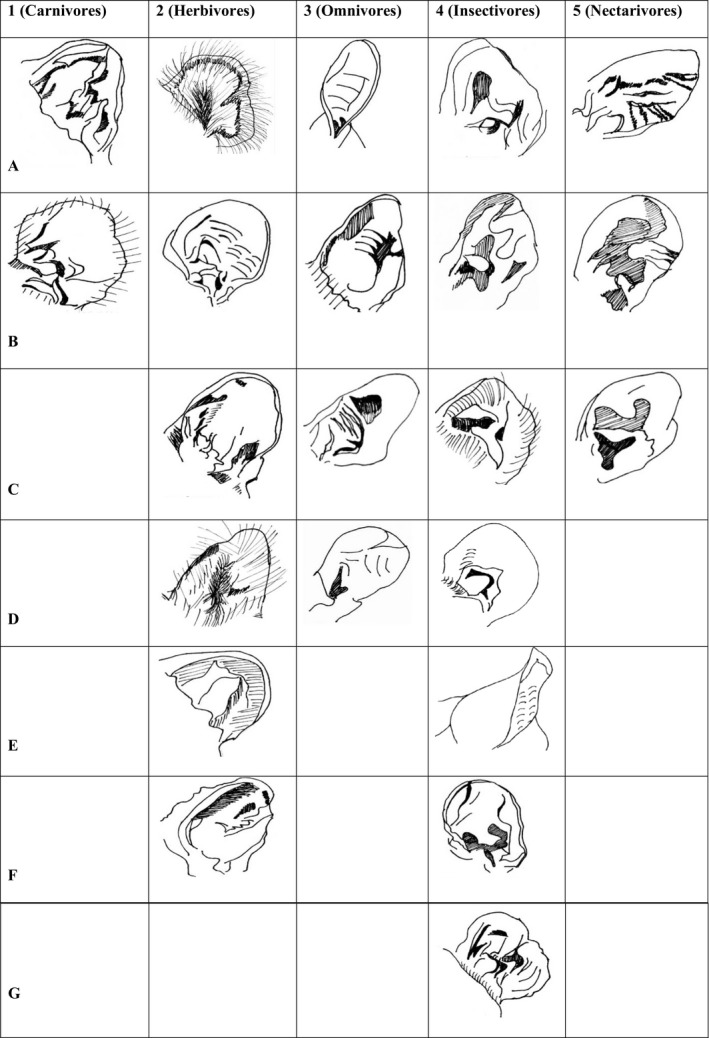
Drawings of the left pinnae of 22 of the focal species categorized into their diet groups. These are not drawn to scale but are instead intended to depict the major folds, shape, and features of each pinna. Carnivores 1A *D. maculatus*, 1B *S. harissii*, Herbivores 2A *P. cinereus*, 2B *P. peregrinus*, 2C *T. stigmatica*, 2D *V. ursinus*, 2E *S. brachyurus*, 2F *T. vulpecula*, Omnivores 3A *M*.* lagotis*, 3B *P. nasuta*, 3C *I. macrourus*, 3D *B. penicillata*, Insectivores 4A *P. tapoatafa*, 4B *P. calura*, 4C *D. cristicaudata*, 4D *A. laniger*, 4E *D. trivirgata*, 4F *S. crassicaudata*, 4G *A. flavipes*, Nectarivores 5A *P. breviceps*, 5B *T. rostratus*, 5C *A. pygmaeus*

Ears from 23 species were measured (Table 1) and described. Across all the species studied the honey possum (*Tarsipes rostratus*) had the shortest scapha ($\bar{x}$ = 8.4 mm) while the bilby (*Macrotis lagotis*) had the longest ($\bar{x}$ = 80.5 mm). The honey possum had the narrowest scapha width ($\bar{x}$ = 7.0 mm) while the koala (*Phascolarctos cinereus*) had the widest ($\bar{x}$ = 41.8mm). Tragi were smallest in the honey possum ($\bar{x}$ = 2.7 mm) and largest in the Tasmanian devil (*Sarcophilus harrissi*) ($\bar{x}$ = 7.8 mm). Interaural distance was smallest in the honey possum ($\bar{x}$ = 5.2 mm) and largest in the Tasmanian devil ($\bar{x}$ = 96.4 mm). Figure 1 provides a morphological comparison of all ears examined.

**TABLE 1:** Diet category, targus presence and mean ± *SD* percentage of body length of measured ear features (scapha length, scapha width, tragus width and interaural distance)

Species	Scientific name	*N*	Diet category	Tragus present	% body length			
					Scapha length	Scapha width	Tragus width	Interaural distance
Dasyuromorphia								
Fat‐tailed dunnart	*Sminthopsis crassicaudata*	4	I	Y	25.2 ± 2.6	20.4 ± 2.3	4.4 ± 1.2 (3)	16.8 ± 3.5
Kultarr	*Antechinomys laniger*	4	I	Y	18.1 ± 1.1	13.6 ± 1.1	3.5 ± 0.6	12.5 ± 1.4
Yellow‐footed antechinus	*Antechinus flavipes*	4	I	Y	13.9 ± 2.0	13.5 ± 3.2	3.5 ± 0.4 (3)	16.6 ± 5.7
Red‐tailed phascogale	*Phascogale calura*	4	I	Y	14.0 ± 2.9	15.9 ± 4.0	3.8 ± 0.3	14.8 ± 2.7
Brush‐tailed phascogale	*Phascogale tapoatafa*	4	I	Y	9.8 ± 2.6	9.4 ± 3.4	2.3 ± 0.6 (3)	11.5 ± 2.9
Crest‐tailed mulgara	*Dasycercus cristicauda*	4	I	Y	10.3 ± 1.8	10.5 ± 0.9	3.1 ± 0.4	12.5 ± 2.4
Spotted‐tailed quoll	*Dasyurus maculatus*	4	C	Y	5.9 ± 2.3	5.2 ± 2.0	1.5 ± 0.2 (3)	9.4 ± 2.3
Tasmanian devil	*Sarcophilus harrisii*	3	C	Y	12.3 ± 1.5	13.1 ± 0.4	2.6 ± 1.0	30.5 ± 1.6
Numbat	*Myrmecobius fasciatus*	4	I	N	8.9 ± 0.7	4.3 ± 0.8		9.2 ± 0.4
Diprotodontia								
Feathertail glider	*Acrobates pygmaeus*	11	N	Y	12.8 ± 3.8	12.2 ± 6.7	2.6 ± 2.9 (6)	17.9 ± 3.4
Honey possum	*Tarsipes rostradus*	4	N	Y	12.9 ± 5.0	10.8 ± 3.6	4.1 ± 1.7 (2)	7.7 ± 0.4
Sugar glider	*Petaurus breviceps*	20	N	N	11.2 ± 2.7	8.7 ± 1.6		15.3 ± 3.2
Striped possum	*Dactylopsila trivirgata*	4	I	N	7.4 ± 0.9	6.5 ± 1.6		18.8 ± 3.0
Common ringtail possum	*Pseudocheirus peregrinus*	20	H	N	8.7 ± 2.5	6.7 ± 1.4		9.7 ± 2.2
Brushtail possum	*Trichosurus vulpecula*	4	H	N	9.7 ± 2.3	6.4 ± 1.4		11.0 ± 1.9
Red‐legged pademelon	*Thylogale stigmatica*	4	H	N	9.1 ± 0.7	4.3 ± 0.7		3.0 ± 0.8
Woylie	*Bettongia penicillata*	4	O	N	9.0 ± 1.2	7.0 ± 0.7		7.5 ± 1.1
Quokka	*Setonix bracyurus*	4	H	N	5.8 ± 0.3	5.7 ± 0.7		5.6 ± 2.2
Koala	*Phascolarctos cinereus*	4	H	N	7.5 ± 1.1	7.4 ± 0.7		15.5 ± 0.9
Bare‐nosed wombat	*Vombatus ursinus*	4	H	N	6.0 ± 1.1	3.6 ± 0.9		9.3 ± 1.4
Peramelemorphia								
Greater bilby	*Macrotis lagotis*	4	O	N	27.8 ± 4.3	10.0 ± 1.0		8.5 ± 1.9
Long‐nosed bandicoot	*Perameles nasuta*	17	O	N	10.5 ± 1.9	4.1 ± 1.1		5.9 ± 1.6
Northern brown bandicoot	*Isoodon macrourus*	19	O	N	7.3 ± 1.3	5.3 ± 1.0		7.6 ± 1.5

### 3.2 Presence/absence of tragi

**FIGURE 2:**
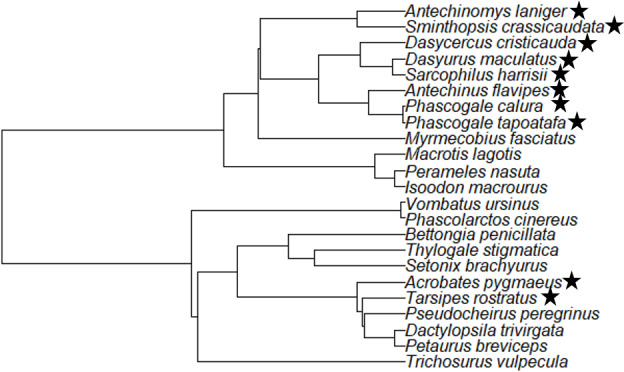
Phylogenetic tree adapted from May‐Collado et al. (2015) showing the relationship between Families and species with (designated by a star) and without tragi (no star)

All species in this study from the Dasyuridae exhibited tragi. The other species examined from the Dasyuromorphia (Myrmecobiidae and Peramelemorphia species) all lacked tragi. Within the Petauroidea examined in this study, the feathertail glider and honey possum had tragi, however, the ringtail possums, striped possum (*Dactylopsila trivirgata*) and sugar glider (*Petaurus breviceps*) lacked tragi. The remainder of the Diprotodontia species included in this study lacked tragi (Table 1). The phylogenetic relationship between species and presence/absence of tragi is shown in Figure 2.

### 3.3 Allometry

The fat‐tailed dunnart (*Sminthopsis crassicaudata*) had the highest tragi relative to body length at 4.4%, followed by the honey possum (4.1%; Table 1). Among *Phascogale* species, the red‐tailed phascogale (*P. calura*) had a larger tragus as a percentage of body length (3.8%) compared to the brush‐tailed phascogale (*P. tapoatafa*; 2.3%). The spotted‐tailed quoll (*Dasyurus maculatus*) had the smallest relative length of tragi to body length (1.5%; Table 1).

Interaural distance in relation to body length range from 3.0% to 30.5% in the marsupials studied. The red‐legged pademelon (*Thylogale stigmatica*) had the smallest interaural percentage, and the Tasmanian devil had the largest (Table 1).

**FIGURE 3:**
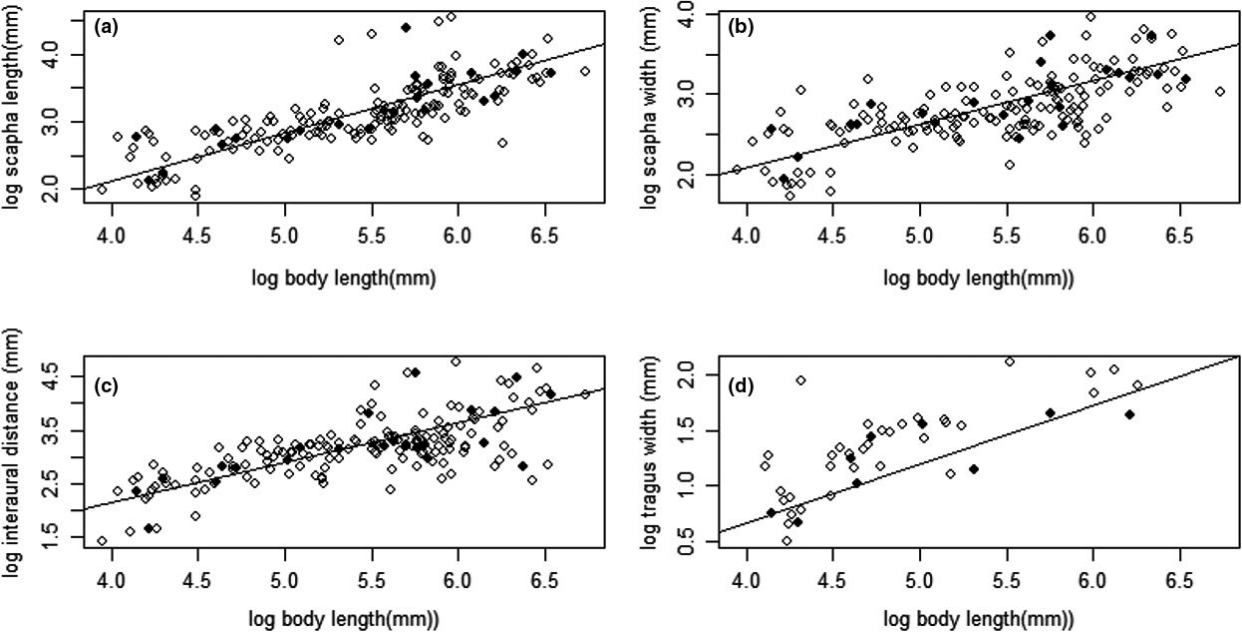
Correlation between body mass of marsupials and (a) scapha length, (b) scapha width, (c) interaural length, and (d) tragus width. Filled in circles are species means, and empty circles are individual data points

For all the marsupials studied, there was a significant positive correlation between body length and scapha length (Table 2; Figure 3a), and between body length and scapha width (Table 2; Figure 3b). There was no significant positive correlation between body length and interaural distance for all marsupials (Table 2; Figure 3c). For the 10 species with tragi present, there was a significant positive correlation between tragus width and body length (Table 2; Figure 3d).

**TABLE 2:** Generalized least squares (PGLS) model results for measure against body length using log‐transformed data

	*n*	Value	Standard error	*t*‐value	*p*‐value
Scapha length	148	0.7122	0.1827	3.8976	**.0008**
Scapha width	148	0.5490	0.2529	2.1705	**.0416**
Interaural distance	148	0.7398	0.4004	1.8476	.0788
Tragus width	35	0.5282	0.1437	3.6750	**.0063**

Note

Significant (<.05) *p*‐values are in bold.

For the dasyurid species, the size of the tragi in relation to scapha length was largest in crest‐tailed mulgara (*Dasycercus cristicauda*) and smallest in the Tasmanian devil (Table 3). Of the other two species that had a tragi, the feathertail glider and crest‐tailed mulgara had the largest tragi to scapha width while the honey possum had the largest tragi to scapha length (Table 3).

**TABLE 3:** Tragi width as a percentage (±*SD*) of scapha width and scapha length in marsupials with tragi

Common name	Scientific name	Tragi	
		% scapha width	% scapha length
Dasyuromorphia			
Yellow‐footed antechinus	*Antechinus flavipes*	25.9 ± 6.5	26.2 ± 7.9
Spotted‐tailed quoll	*Dasyurus maculatus*	29.8 ± 13.5	25.4 ± 3.9
Brush‐tailed phascogale	*Phascogale tapoatafa*	21.2 ± 3.6	21.2 ± 1.0
Red‐tailed phascogale	*Phascogale calura*	27.8 ± 6.7	24.8 ± 6.6
Fat‐tailed dunnart	*Sminthopsis crassicaudata*	18.4 ± 6.8	21.3 ± 6.9
Crest‐tailed mulgara	*Dasycercus cristicauda*	30.6 ± 4.3	29.8 ± 3.2
Kultarr	*Antechinomys laniger*	19.5 ± 3.3	25.9 ± 3.4
Tasmanian devil	*Sarcophilus harrisii*	19.5 ± 7.6	19.2 ± 7.0
Diprotodontia			
Feathertail glider	*Acrobates pygmaeus*	30.5 ± 15.6	30.8 ± 9.3
Honey possum	*Tarsipes rostradus*	27.0 ± 0.6	37.2 ± 6.9

### 3.4 Relationship between diet and ear morphology

When comparing the diet of the different marsupial species, all carnivorous marsupials had tragi (Table 1), while all herbivorous and omnivorous species lacked tragi. All except two insectivorous species, the numbat and striped possum, had tragi.

Scapha length to body length ratios was significantly larger in insectivores compared to carnivores, herbivores, and omnivores (*F*_4,147_ = 6.36 *p* < .05). Scapha width to body length ratios was significantly larger in insectivores compared to all other diet groups (*F*_4,147_ = 18.27 *p* < .05). Interaural distance to body length ratios was significantly larger in carnivores compared to herbivores, omnivores, and insectivores; herbivores were significantly smaller than insectivores; and omnivores were significantly smaller than insectivores and nectarivores (*F*_4,147_ = 26.48 *p* < .05). There was no significant difference between the ratio of tragi to scapha length (*F*_2,33_ = 0.10 *p* = .38) and diet group. The ratio of tragi to scapha width was significantly larger in nectarivores compared to insectivores and carnivores (*F*_2,33_ = 4.56 *p* < .05).

## 4 DISCUSSION

In this study, we conducted an investigation to compare the pinnae structure and diet of 23 Australian marsupial species, specifically presence or absence of tragi. For most families, we only had one representative species from each with the exception of the Dasyuridae, Petauridae, Macropodidae, and Peremalidae families. Of all the species, we examined, 10 had tragi. All dasyurid species examined had tragi, and all are either carnivorous or insectivorous (van Dyck & Strahan, 2008). The insectivorous numbat (Friend, 2008), however, lacked tragi. No herbivorous or omnivorous marsupials investigated had tragi. We also found pinnae size had an allometric relationship to body size in the marsupials studied, as body length increased, so did the size of scapha and tragi.

Few studies have morphologically characterized marsupial ears. Johnson and Johnson (1983) described the pinna (scapha) length in bilbies as 81.2 ± 9.1 mm (*n* = 7), which is similar to the measurements in this study (80.5 ± 12.5 mm). Our results for the kultarr were also similar to those reported previously as 24.0 mm (*n* = 59) for live specimens and 15.6 mm (*n* = 47) for preserved specimens (Lidicker & Marlow, 1970). As Lidicker and Marlow (1970) demonstrated, there is a difference in the scapha length measurements between live and preserved specimens. Our measurements may therefore have been impacted by shrinkage as a result of preservation techniques. Future studies could eliminate any potential shrinkage issues by utilizing only live specimens.

The greater bilby exhibits elongated pinnae that are over double the length that they are in width and is often compared to rabbits and hares (Leporidae) due to similarities in their physical appearance and burrowing habits. However, the bilby and Leporidae evolved on separate continents with differing climates and have different diets. The similarity in pinnae shape between the species is therefore most likely a result of positive selective pressure required by these species to avoid predators, as these species both exhibit predator avoidance (Cowan & Bell, 1986).

The fat‐tailed dunnart had the largest tragi in relation to body size and the crest‐tailed mulgara the largest tragi in relation to scapha size. Both of these Dasyurid species are nocturnal and insectivorous (Chen, Dickman, & Thompson, 1998; Morton, 1978). The insectivorous little brown bat (*Myotis lucifugus*) also has a large tragi compared to the size of their pinnae (Anthony & Kunz, 1977; Clare, Barber, Sweeney, Hebert, & Fenton, 2011). In this bat species, the tragus is used for echolocation purposes to determine distance and size of objects within their area, thus providing advantages in locating and capturing prey in the dark (Jones, 2005). The tragus amplifies lower acoustic responses and creates larger sound waves for interpretation by the middle and inner ear (Honda, 1985). While the specifics of how dunnarts and mulgara capture their prey is unknown, Bos (2001) has suggested most small dasyurids engage in ‘foraging walks’ whereby they stop and scan their environment to listen or sniff to locate prey. Pellis and Nelson (1984) suggest that movement and sound made by prey enhanced detection by eastern quolls (*Dasyurus viverrinus*). It may be that like the little brown bat, the tragi of dunnarts enable them to acquire additional advantages in terms of prey location and hence capture.

Insectivorous bats employ echolocation to identify and judge the wingbeat sounds and frequency of their prey (Ratnam, Condon, & Feng, 1996). For example, the big brown bat tragus improves the gain and direction of high‐frequency sounds (60–90 kHz), specifically the acoustics and localization of noise (Koay et al., 1998). Hence, as echolocation is used in conjunction with a tragus, the concept that some marsupial species also use this method to pinpoint their prey is not entirely impractical. Most species in this study categorized as insectivorous had tragi, suggesting their pinnae aid in prey detection. The striped possum and the numbat were the only two species in the insectivorous diet group lacking a tragus, however, neither are dasyurids. While these lack of features in the striped possum and numbat conflict with the idea that tragi aid in prey detection in carnivorous and insectivorous species, it needs to be noted that the striped possum has some unique features compared to other members of the Petauridae family, including shorter body length, stronger bite, and an extended fourth finger. These features appear to be adaptations to assist removal of common prey such as larvae from wood (Rawlins & Handasyde, 2002). Therefore, in the case of the striped possum, that has other specialisations to increase prey capture, a tragus may not be a necessary feature on their pinnae. The numbat is also a specialized termite feeder (Friend, 2008), and it is likely that it does not require the sophisticated tragi anatomy to detect termites residing in termite mounds during the day, when compared to other species needing to locate more mobile and hidden invertebrates at night.

No herbivorous or omnivorous species displayed tragi. Species examined within these dietary groups also had simpler pinna structure, specifically less folds in the concha structure, when compared to insectivorous, carnivorous, and nectarivorous species (Figure 1). One example in this study is the common ringtail possum (*Pseudocheirus peregrinus*), an arboreal species with a primarily herbivorous diet of eucalypt foliage (Chilcott & Hume, 1985; Hermsen, Kerle, & Old, 2016). Common ringtail possums often fall prey to higher trophic level species such as the introduced European red fox (*Vulpes vulpes*) and powerful owls (*Ninox strenua*) (Kavanagh, 2002; White, Gubiani, Smallman, Snell, & Morton, 2006). These possums, however, display strong predatory avoidance when presented with olfactory cues, rather than auditory cues (Anson & Dickman, 2013), hence likely do not require complexity in their pinnae structure (and tragi).

In contrast, two of the three species in the nectarivorous group displayed tragi. These species (honey possum and feathertail glider) were gathered together in this dietary group due to their similar dietary requirements of honey, sap, pollen, and nectar, however, the sugar glider and feathertail glider also consume a small amount of insects (Bradshaw & Bradshaw, 2001; Herrmann et al., 2013). The honey possum is the only known marsupial to live exclusively on nectar and pollen. Its mouth and skull are believed to have evolved morphologically as a consequence of the species' relationship with the native plant genera to feed on this specialized diet (Smith, 1982). It is therefore unlikely that the nectarivores have developed tragi as a specialized portion of their pinnae to detect prey, as their diet does not rely heavily on insects. It is more reasonable to assume they have developed this feature as a function to aid in hearing predators to avoid being consumed.

We found there was a positive correlation between external ear anatomy and body size of marsupials. The Tasmanian devil had the largest interaural distance, and this is not unexpected as generally a larger distance between the two ears commonly results in an allometric scaling of larger pinnae. Greater interaural distances have been correlated with accuracy in sound localization and appear more effective when compared to smaller species with closer set ears. Species, including marsupials, with smaller interaural distances, however, hear higher frequency sounds (Nichols, Heffner, & Heffner, 1981; Old et al., 2020).

The red‐legged pademelon displayed the smallest interaural distance in relation to body length. While no studies have been conducted on interaural distance of this species previously, Blumstein, Daniel, Griffin, and Evans (2000) conducted a study of the tammar wallaby (*Notamacropus eugenii*), a species likely to have a similar acoustic requirement to the red‐legged pademelon. However, pinnae development in prey species, such as macropods, may not have evolved entirely for hearing specific predatory noises, as predators often adopt a silent hunting mechanism (Apfelbach et al., 2005). Research instead suggests that the auditory ability of a prey species is based on hearing conspecifics who send a warning signal once a predator is viewed (Blumstein et al., 2000). Specifically, Blumstein et al. (2000) demonstrated that the tammar wallaby was not significantly responsive to predator noise (*Canis lupus dingo* howls and *Aquila audax* calls), however, did display decreased foraging and increased cautionary behavior, such as looking around, in response to foot‐thumping sounds, a commonly used predator alarm signal in wallabies. Wallabies also displayed stronger responses to visual stimuli of predators compared to predator noise (Blumstein et al., 2000). These findings suggest that species with a smaller interaural distance in relation to body length, such as wallabies, rely less on sound localization of noise origin, and more on the presence and type of sound they hear to guide them in their next action.

Aside from one case where a human subject was able to manipulate their auricular muscles in such a way as to move their tragi voluntarily (Neame, 1988), the tragus is regarded as an immobile part of the pinna. However, mobility in the scapha of the pinna has been recorded in some mammalian species such as the big brown bat (Aytekin et al., 2004). Aitkin (1997) suggests that nocturnal animals with mobile pinnae lack enlarged prominent tragi as they can compensate for the ability of the tragus by moving their ears to pinpoint sounds and focus on specific noises. This idea has been supported by Johnson and Johnson (1983) for the greater bilby that has independently mobile pinnae, having noted their ears remained erect after emerging from their burrow. Early detection of a predator would be advantageous as it provides additional time for an animal to escape, however, the relationship between pinna size and tragus would benefit from further investigations into which marsupials are able to move their pinnae consciously. If mobile pinnae aid predator detection, it may explain an absence of specified pinnae features, such as a tragus, in some herbivorous species.

## 5 CONCLUSIONS

This study indicates that there is a possible correlation between the pinnae of Australian marsupial species and their diet. Furthermore, the size and features of marsupial pinnae are therefore likely to be a strong indicator of their acoustic biotope requirements. No herbivores investigated in this study exhibited tragi, and hence, it is likely that this pinnae feature is not required for their survival. However, the more complex pinnae with tragi were mainly associated with carnivorous, insectivorous, and nectarivorous species suggesting that the tragi in these species likely benefits prey capture and consequently their survival. A future study incorporating a larger number of individuals of each species, including some from Papua New Guinea and South America, would be advantageous.

## CONFLICT OF INTEREST

The authors have no competing interests.

## AUTHOR CONTRIBUTIONS

**Hayley J. Stannard:** Data curation (equal); formal analysis (equal); methodology (equal); validation (equal); visualization (equal); writing – review and editing (equal). **Kathryn Dennington:** Investigation (equal); writing – original draft (equal). **Julie M. Old:** Conceptualization (equal); formal analysis (equal); investigation (equal); methodology (equal); project administration (equal); supervision (equal); validation (equal); writing – original draft (equal); writing – review and editing (equal).

## Data Availability

All data are available within this paper.

## APPENDIX 1

Specimen measurements of pinnae features and Australian Museum identification number.

**Table:**

Genus	Species	Museum ID	Sex	Scapha length (mm)	Scapha width (mm)	Tragus width (mm)	Interaural distance (mm)	Body length (mm)
*Acrobates*	*pygmaeus*	M2615	F	9.04	7.44		14.81	73.80
*Acrobates*	*pygmaeus*	M3294	F	8.01	6.66		13.52	63.75
*Acrobates*	*pygmaeus*	M4109	M	7.20	7.53	2.47	12.87	89.00
*Acrobates*	*pygmaeus*	M5072	M	8.57	5.61	2.44	11.33	70.52
*Acrobates*	*pygmaeus*	M5378	M	7.60	6.41	1.66	10.82	69.17
*Acrobates*	*pygmaeus*	M6151	F	8.29	6.51	2.20	12.18	75.00
*Acrobates*	*pygmaeus*	M8172	F	8.55	7.40		11.90	79.00
*Acrobates*	*pygmaeus*			11.83	21.13	6.98	33.45	74.88
*Acrobates*	*pygmaeus*			14.79	12.34	1.94	17.15	69.53
*Antechinomys*	*laniger*	M588A	F	17.50	13.63	3.23	10.20	89.00
*Antechinomys*	*laniger*	M8428	M	20.69	15.29	3.22	17.34	119.00
*Antechinomys*	*laniger*	M8463	F	16.34	12.29	3.83	10.91	94.00
*Antechinomys*	*laniger*	M8464	M	17.67	13.13	3.63	12.28	98.80
*Antechinus*	*flavipes*	591.00	F	12.99	10.91	0.00	14.76	96.48
*Antechinus*	*flavipes*	M11514	M	11.52	13.37	3.56	16.24	90.00
*Antechinus*	*flavipes*	M2886	F	15.55	12.99	4.40	12.20	125.53
*Antechinus*	*flavipes*	M5375	M	17.12	17.67	3.19	23.75	102.00
*Bettongia*	*penicillata*	M3090	F	24.94	21.56	0.00	29.17	338.00
*Bettongia*	*penicillata*	M42682	F	32.50	20.97	0.00	21.12	328.00
*Bettongia*	*penicillata*	M42683	M	31.48	24.80	0.00	26.27	320.00
*Bettongia*	*penicillata*	M45755	M	25.43	22.23	0.00	19.33	293.00
*Dactylopsila*	*trivirgata*	M38910	M	17.94	14.21	0.00	37.23	230.00
*Dactylopsila*	*trivirgata*	M41272	F	15.31	11.66	0.00	53.93	245.00
*Dactylopsila*	*trivirgata*	M42750	M	21.61	16.17	0.00	42.66	260.00
*Dactylopsila*	*trivirgata*	M42751	F	16.61	19.89	0.00	47.50	230.00
*Dasycercus*	*cristicaudata*	M2987	M	19.51	15.80	4.93	19.90	172.00
*Dasycercus*	*cristicaudata*	M8638	F	11.67	16.56	4.15	14.57	153.00
*Dasycercus*	*cristicaudata*	M8639	F	16.59	15.73	4.97	21.97	147.00
*Dasycercus*	*cristicaudata*	M8641	M	14.80	14.97	4.70	18.50	134.00
*Dasyurus*	*maculatus*	M6637	F	14.70	23.20	6.72	38.71	523.00
*Dasyurus*	*maculatus*	M7646	M	37.92	30.27	7.64	46.25	456.00
*Dasyurus*	*maculatus*	M8048	F	26.20	28.10	6.20	50.48	408.00
*Dasyurus*	*maculatus*	M9069	M	37.50	17.00	0.00	48.00	620.00
*Isodoon*	*macrourus*	M10371	M	29.69	22.46	0.00	41.28	439.00
*Isodoon*	*macrourus*	M10742	M	29.54	20.66	0.00	24.45	422.50
*Isodoon*	*macrourus*	M10755	M	30.77	17.28	0.00	34.67	382.00
*Isodoon*	*macrourus*	M18532	M	25.38	16.94	0.00	33.42	362.00
*Isodoon*	*macrourus*	M25001	F	19.19	12.78	0.00	26.92	251.00
*Isodoon*	*macrourus*	M25003	M	25.10	16.21	0.00	21.70	355.50
*Isodoon*	*macrourus*	M26351	M	23.55	16.88	0.00	20.97	253.00
*Isodoon*	*macrourus*	M8181	F	30.41	14.74	0.00	31.29	436.00
*Isodoon*	*macrourus*	M8230	M	27.21	31.32	0.00	34.65	511.00
*Isodoon*	*macrourus*	M8408	F	16.21	13.63	0.00	13.39	184.00
*Isodoon*	*macrourus*	M9142	F	18.29	11.77	0.00	13.82	185.00
*Isodoon*	*macrourus*	M9419	M	23.73	17.06	0.00	25.05	300.00
*Isodoon*	*macrourus*	M9420	M	23.11	13.66	0.00	22.37	289.00
*Isodoon*	*macrourus*	M9767	F	25.81	19.18	0.00	28.22	352.00
*Isoodon*	*macrourus*	M34982	M	17.58	16.72	0.00	15.67	275.00
*Isoodon*	*macrourus*	M47932	M	15.16	15.00	0.00	22.67	340.00
*Isoodon*	*macrourus*	M8168	F	22.79	14.45	0.00	18.26	350.00
*Macrotis*	*lagotis*	M26831	M	87.64	30.99	0.00	24.50	360.00
*Macrotis*	*lagotis*	M27340	M	93.82	41.86	0.00	27.08	390.00
*Macrotis*	*lagotis*	M5337	F	73.85	24.90	0.00	26.24	245.00
*Macrotis*	*lagotis*	M8620	F	66.60	21.80	0.00	19.58	204.00
*Myrmecobius*	*fasciatus*	M1429	F	25.31	13.39	0.00	24.28	263.00
*Myrmecobius*	*fasciatus*	M1999	M	22.43	8.28	0.00	21.56	251.00
*Myrmecobius*	*fasciatus*	M2000	F	20.76	11.78	0.00	23.97	260.00
*Myrmecobius*	*fasciatus*	M3102	F	26.21	12.54	0.00	27.54	285.00
*Perameles*	*nasuta*	M2872	F	37.31	19.60	0.00	26.20	385.00
*Perameles*	*nasuta*	M2873	F	26.20	11.06	0.00	16.18	188.00
*Perameles*	*nasuta*	M2874	F	39.36	14.11	0.00	17.67	372.00
*Perameles*	*nasuta*	M3389	M	44.84	13.90	0.00	13.45	367.00
*Perameles*	*nasuta*	M3712	F	39.28	12.04	0.00	14.52	388.00
*Perameles*	*nasuta*	M3734	M	24.26	11.33	0.00	12.21	186.10
*Perameles*	*nasuta*	M4274	F	35.23	11.90	0.00	16.58	319.00
*Perameles*	*nasuta*	M5152	M	32.44	12.87	0.00	21.87	418.50
*Perameles*	*nasuta*	M5153	F	37.38	16.12	0.00	30.49	370.00
*Perameles*	*nasuta*	M6842	F	34.54	10.78	0.00	22.99	385.50
*Perameles*	*nasuta*	M6882	M	31.58	13.22	0.00	22.36	371.00
*Petaurus*	*breviceps*	M19568	M	17.26	13.12	0.00	24.78	181.00
*Petaurus*	*breviceps*	M19976	F	21.60	14.57	0.00	17.00	163.00
*Petaurus*	*breviceps*	M20387	M	16.53	12.04	0.00	25.32	172.00
*Petaurus*	*breviceps*	M20526	F	16.43	12.69	0.00	21.52	155.00
*Petaurus*	*breviceps*	M26642	M	13.04	13.29	0.00	26.76	128.46
*Petaurus*	*breviceps*	M26647	F	20.21	12.95	0.00	20.95	165.00
*Petaurus*	*breviceps*	M29253	F	17.03	11.12	0.00	22.20	130.04
*Petaurus*	*breviceps*	M30018	F	17.52	13.34	0.00	21.56	154.10
*Petaurus*	*breviceps*	M30019	M	15.22	12.58	0.00	22.33	142.89
*Petaurus*	*breviceps*	M30020	M	12.90	10.16	0.00	27.11	143.13
*Petaurus*	*breviceps*	M30680	M	20.31	12.35	0.00	20.24	139.00
*Petaurus*	*breviceps*	M30682	F	16.33	12.55	0.00	23.54	117.00
*Petaurus*	*breviceps*	M30683	M	20.25	13.40	0.00	20.75	135.00
*Petaurus*	*breviceps*	M30684	F	17.88	13.81	0.00	26.51	120.08
*Petaurus*	*breviceps*	M32569	M	19.08	15.66	0.00	27.29	182.00
*Petaurus*	*breviceps*	M32642	F	24.47	19.74	0.00	25.18	158.00
*Petaurus*	*breviceps*	M5077	M	15.69	18.14	0.00	30.12	195.00
*Petaurus*	*breviceps*	M5307	F	17.92	15.89	0.00	23.10	214.00
*Petaurus*	*breviceps*	M5406	M	15.69	14.72	0.00	26.82	225.00
*Petaurus*	*breviceps*	M5734	F	16.59	14.90	0.00	27.23	223.00
*Phascogale*	*calura*	M44879	M	14.34	16.89	4.44	13.87	120.00
*Phascogale*	*calura*	M44880	F	14.79	15.46	3.77	19.45	107.00
*Phascogale*	*calura*	M44881	F	13.35	14.51	4.69	15.72	110.60
*Phascogale*	*calura*	M44883	M	20.01	24.23	3.94	16.80	110.80
*Phascogale*	*tapoatafa*	M37468	M	17.03	14.88	3.00	13.96	178.50
*Phascogale*	*tapoatafa*	M43405	F	18.83	21.81	4.68	24.45	190.00
*Phascogale*	*tapoatafa*	M44901	F	22.73	21.75	4.81	25.04	173.00
*Phascogale*	*tapoatafa*	M8278	M	18.29	13.78	0.00	28.98	272.00
*Phascolarctos*	*cinereus*	M23795	M	38.50	42.41	0.00	103.46	635.00
*Phascolarctos*	*cinereus*	M23796	F	40.81	44.73	0.00	77.05	540.00
*Phascolarctos*	*cinereus*	M24515	M	48.48	40.11	0.00	86.73	565.00
*Phascolarctos*	*cinereus*	M6805	F	40.96	39.96	0.00	83.55	520.00
*Pseudocheirus*	*peregrinus*	M390089	F	26.66	20.50	0.00	25.11	255.00
*Pseudocheirus*	*peregrinus*	M39380	M	30.94	21.65	0.00	21.79	330.00
*Pseudocheirus*	*peregrinus*	M46647	M	16.10	18.44	0.00	44.47	330.00
*Pseudocheirus*	*peregrinus*	M46648	F	20.21	17.90	0.00	31.53	270.00
*Pseudocherius*	*peregrinus*	M23581	M	32.36	20.68	0.00	24.44	311.00
*Pseudocherius*	*peregrinus*	M24348	M	19.74	22.69	0.00	26.62	320.00
*Pseudocherius*	*peregrinus*	M30182	F	20.76	23.22	0.00	28.71	280.00
*Pseudocherius*	*peregrinus*	M32568	F	22.64	14.05	0.00	26.03	204.00
*Pseudocherius*	*peregrinus*	M33364	M	31.91	23.09	0.00	32.59	316.00
*Pseudocherius*	*peregrinus*	M33365	F	22.63	14.57	0.00	10.91	273.00
*Pseudocherius*	*peregrinus*	M33366	M	34.27	20.58	0.00	27.20	320.00
*Pseudocherius*	*peregrinus*	M33367	F	20.84	21.19	0.00	28.60	375.00
*Pseudocherius*	*peregrinus*	M33368	F	22.66	18.81	0.00	27.87	329.00
*Pseudocherius*	*peregrinus*	M33369	F	22.50	16.72	0.00	30.91	317.00
*Pseudocherius*	*peregrinus*	M33370	F	20.49	13.60	0.00	29.05	276.00
*Pseudocherius*	*peregrinus*	M33371	M	17.75	17.41	0.00	22.83	246.00
*Pseudocherius*	*peregrinus*	M35236	F	20.26	15.18	0.00	19.45	193.00
*Pseudocherius*	*peregrinus*	M35237	M	20.07	14.96	0.00	23.66	191.00
*Pseudocherius*	*peregrinus*	M35445	F	21.20	13.30	0.00	28.58	297.00
*Pseudocherius*	*peregrinus*	M39384	M	21.55	14.63	0.00	15.99	137.00
*Sarcophilus*	*harrisii*	41383.00	F	31.69	38.44	0.00	97.00	302.00
*Sarcophilus*	*harrisii*	45865.00	F	32.88	33.86	8.20	76.18	250.00
*Sarcophilus*	*harrisii*	M47935	M	52.55	52.11	7.44	116.00	400.00
*Setonix*	*bracyurus*	M3083	M	22.90	27.84	0.00	23.96	420.00
*Setonix*	*bracyurus*	M3086	F	23.80	23.93	0.00	35.33	410.00
*Setonix*	*bracyurus*	M3087	F	30.90	26.54	0.00	24.52	528.00
*Setonix*	*bracyurus*	M9056	M	31.87	26.53	0.00	18.57	515.00
*Sminthopsis*	*crassicaudata*	M8243	F	16.83	12.81	2.38	9.73	68.00
*Sminthopsis*	*crassicaudata*	M8323	F	17.48	15.92	2.57	9.12	67.00
*Sminthopsis*	*crassicaudata*	M8503	M	13.53	12.19	3.55	12.87	62.00
*Sminthopsis*	*crassicaudata*	M9836	M	15.98	10.97		10.67	57.00
*Tarsipes*	*rostradus*	M3128	F	6.69	5.90		6.56	89.00
*Tarsipes*	*rostradus*	M3311	M	7.20	7.73		4.20	52.00
*Tarsipes*	*rostradus*	M33966	M	11.76	7.68	3.23	4.90	61.00
*Tarsipes*	*rostradus*	M4509	M	7.90	6.50	2.10	5.30	71.00
*Thylogale*	*stigmatica*	M5640	M	54.10	21.34	0.00	12.77	620.00
*Thylogale*	*stigmatica*	M5641	F	47.05	20.41	0.00	16.90	500.00
*Thylogale*	*stigmatica*	M5643	F	46.50	25.48	0.00	21.20	550.00
*Thylogale*	*stigmatica*	M7347	M	67.96	34.23	0.00	17.13	680.00
*Trichosurus*	*vulpecula*	M42736	F	45.06	24.50	0.00	38.70	360.00
*Trichosurus*	*vulpecula*	M45866	M	41.20	31.10	0.00	52.80	390.00
*Trichosurus*	*vulpecula*	M45867	F	38.15	26.28	0.00	40.29	445.00
*Trichosurus*	*vulpecula*	M48184	M	40.06	26.67	0.00	59.70	557.00
*Vombatus*	*ursinus*	M1479	F	45.80	27.80	0.00	54.50	610.00
*Vombatus*	*ursinus*	M1537	F	42.04	20.70	0.00	62.97	839.00
*Vombatus*	*ursinus*	M3422	M	41.63	21.86	0.00	70.78	670.00
*Vombatus*	*ursinus*	M42800	M	35.51	26.41	0.00	66.73	650.00

NA means the tragus were nonexistent on either ear of the specimen.
